# Does an Invasive Bivalve Outperform Its Native Congener in a Heat Wave Scenario? A Laboratory Study Case with *Ruditapes decussatus* and *R. philippinarum*

**DOI:** 10.3390/biology10121284

**Published:** 2021-12-07

**Authors:** Daniel Crespo, Sara Leston, Lénia D. Rato, Filipe Martinho, Sara C. Novais, Miguel A. Pardal, Marco F. L. Lemos

**Affiliations:** 1 MARE—Marine and Environmental Sciences Centre, ESTM, Polytechnic of Leiria, 2520-641 Peniche, Portugal; lenia.rato@ipleiria.pt (L.D.R.); sara.novais@ipleiria.pt (S.C.N.); 2 CFE—Centre for Functional Ecology—Science for People & the Planet, Department of Life Sciences, University of Coimbra, Calçada Martim de Freitas, 3000-456 Coimbra, Portugal; saraleston@ci.uc.pt (S.L.); fmdm@ci.uc.pt (F.M.); mpardal@uc.pt (M.A.P.); 3 CIIMAR—Interdisciplinary Centre of Marine and Environmental Research, University of Porto, Terminal de Cruzeiros do Porto de Leixões, Avenida General Norton de Matos, S/N, 4450-208 Matosinhos, Portugal

**Keywords:** biological invasions, biomarkers, bioturbation, climate change, ecosystem functioning, experimental ecology, integrated biological response

## Abstract

**Simple Summary:**

Global climate change is responsible for more frequent heat waves, which offers opportunities for invasive species to expand their range. Two congener bivalves, the native *Ruditapes decussatus* and the invasive *R. philippinarum*, were exposed to a heat wave aquaria simulation and analysed for ecological and subcellular biomarkers responses. Despite reduced responses on the ecological level (bioturbation and nutrient concentration), there were differential responses to the heat wave at the subcellular level, where the invasive species seems to be less impacted than the native by the heat wave. This reinforces the common notion that climate change events may provide opportunities for biological invasions.

**Abstract:**

Global warming and the subsequent increase in the frequency of temperature anomalies are expected to affect marine and estuarine species’ population dynamics, latitudinal distribution, and fitness, allowing non-native opportunistic species to invade and thrive in new geographical areas. Bivalves represent a significant percentage of the benthic biomass in marine ecosystems worldwide, often with commercial interest, while mediating fundamental ecological processes. To understand how these temperature anomalies contribute to the success (or not) of biological invasions, two closely related species, the native *Ruditapes decussatus* and the introduced *R. philippinarum*, were exposed to a simulated heat wave. Organisms of both species were exposed to mean summer temperature (~18 °C) for 6 days, followed by 6 days of simulated heat wave conditions (~22 °C). Both species were analysed for key ecological processes such as bioturbation and nutrient generation—which are significant proxies for benthic function and habitat quality—and subcellular biomarkers—oxidative stress and damage, and energetic metabolism. Results showed subcellular responses to heat waves. However, such responses were not expressed at the addressed ecological levels. The subcellular responses to the heat wave in the invasive *R. philippinarum* pinpoint less damage and higher cellular energy allocation to cope with thermal stress, which may further improve its fitness and thus invasiveness behaviour.

## 1. Introduction

Invasive species (IS) are recognized as a worldwide nuisance [1,2] and are often reported as able to occupy niches that were emptied due to abiotic pressures, such as pollutants [3,4], floods, heat waves or droughts [5,6], warming, changes in hydric regimen and habitat modification [7]. Some species are deliberately introduced for their economic value as food source in an aquaculture context [8,9]. Invasive species often modify existing ecological processes, which imply a shift on the ecosystem functioning [1,2], due to the introduction of new traits within the native community and/or the reduction of native traits. Nevertheless, context, i.e., the receiving habitat characteristics, as well as the timing of invasive events, or other undisclosed biotic and abiotic determinants, define the success and impacts of IS [10,11].

Global climate change is responsible not only for the increase in the average atmospheric temperature but also has implications in short-duration events such as droughts, floods, and thermal anomalies such as cold spells or heat [12]. Particularly, heat waves are expected to become longer, more severe, and more frequent in the next decades [6,12]. In the Iberian Peninsula between 1979 and 2017, the number of heat wave events in each year have been increasing on average by 1 per decade, while the number of days under a heat wave in each year is increasing at the rate of 2.5 days per decade, with the highest number of heat waves (10) occurring in 2003 and 2017 [13]. Under the worst scenario of greenhouse gas emissions, some climatic models predict that by 2100 all the oceans will be close to a permanent state of heat wave, i.e., 365 days per year [14]. There is evidence that marine heat waves (MHW) interfere with biodiversity and ecosystem functioning [15,16], threatening the provision of relevant ecosystem services [6]. Despite the existence of multiple definitions, a heat wave is usually described as an extended period (usually 5 or more days) when temperatures are higher than the typical values for the same period in other years [6,16]. The effects of MHW can be especially strong among species that live close to the upper limit of their thermal tolerance, and among those with reduced mobility [6]. Simultaneously, it is known that species and biological communities suffer stronger impacts under sudden, short duration climatic events, when compared with slower changes on average conditions [15,17,18]. Therefore, introduced non-native species with wider thermal tolerance ranges, or higher thermal thresholds, may benefit from MHW to outcompete their native counterparts [19]. 

Understanding and predicting the interactive effects between invasions and climate change implies a stepwise approach. Researchers should start by understanding if non-native invasive species and native species respond differentially to climate events [4,20]. There is a plethora of choices for tools to address such a question, which should be answered by a multilevel, integrative approach, ranging from ecological to subcellular levels of biological organization [21]. Such an integrative multiple levels approach includes ecological relevant endpoints and biomarkers that may be linked to population impacts potentially seen later in time [21]. This will allow disclosure of some of the underlying effects of these heat waves which may result in different trade-offs and fitness costs for these species. If IS have reduced trade-offs and fitness costs, this could be one of the drivers that increase the ability to invade and thrive over other species [22,23]. This multilevel analysis also contributes to understanding whether the effects of these climate stressors propagate from lower to higher levels of biological organization.

The analysis of sediment reworking—bioturbation—and nutrient concentrations in the water column (respectively an ecosystem process and an ecosystem function [18]) are relevant proxies for faunal activities and behaviour [22,24,25], by exposing both population and individual responses to different environmental stimulus. Bioturbation, as the passive and active displacement of sediment particles by fauna, is a fundamental process that mediates carbon remineralization, nutrient dynamics across the sediment–water interface, and sediment aeration, bioavailability, and ultimately intervenes in biological production and ecological health of ecosystems [24,26,27]. Simultaneously, the use of subcellular biomarkers, namely those related to oxidative stress and damage, and energy metabolism, provide finer detail on the effects of environmental stressors, such as temperature [5], salinity [23], or contaminants and pathogens [28], on individual metabolic responses. These reflect the internal individual environment, and are endpoints that allow the assessment of fast responses to external drivers, even under less intense stressor levels [21,29], being systematically applied in ecotoxicology. By addressing both lower and higher levels of biological organization responses, researchers can spot some of the present impacts or later trade-offs that may occur in both native and non-native species under different environmental scenarios [23]. Although this question is fundamental to understand the success or failure of non-native invasive species, there is a consistent lack of literature in which both native and non-native closely related species are compared under similar scenario exposures [30].

The Manila clam *Ruditapes philippinarum* (A. Adams & Reeve, 1850) has its origins in the Indo-Pacific coasts, and due to its worldwide expansion, high fecundity, and growth rates, is featured among the most relevant coastal IS. It lives under a wide range of environmental conditions, between 14 and 42 PSU [23], and 6 to 30 °C [31], as well as in different types of habitat (coastal mid-tidal levels, coastal lagoons, or estuaries) [32]. The Manilla clam was introduced in Portugal for aquaculture and harvesting purposes (first reported in 1984 [33], probably as secondary introduction from Spanish populations [34]), after the decline in native populations of grooved carpet shell *R. decussatus* (Linnaeus, 1758) and pullet carpet shell *Venerupis corrugata* (Gmelin, 1791), and cultured oyster stocks [32,33]. Worldwide, in 2014, *R. philippinarum* accounted for 25% of total mollusc production [31]. While not licensed for aquaculture production in Portugal, the species has been actively dispersed by harvesters throughout several estuaries [33], and is often a dominant species in invasion sites. It is a highly valued species: in 2018, the *R. philippinarum* official production in Portugal accounted for 862.3 tons/€ 1,501,350.53 [35]. The native congener *R. decussatus* shares its ecological niche with the invasive *R. philippinarum*, which overlaps with 40% of the former’s trophic niche [32]. *Ruditapes decussatus* is widely used in bivalve aquaculture and is distributed along the European Atlantic and Mediterranean coasts [36]. It shows a wider salinity tolerance than *R. philippinarum*, between 7 and 42 PSU [23], but seems to be less sensitive to high temperatures [37]. Both species have been successfully used as biological monitors for coastal contamination and environmental quality [23,38].

Biomarkers are sub-individual endpoints that are recognized for giving potential responses on effects of a given stress, earlier-in-time than the other endpoints measured on the population or community [21]. These may include oxidative stress and damage endpoints, as stress induces the increase of mitochondrial respiration and of reactive oxygen species (ROS), that have the ability to damage lipids, DNA, and proteins [39]. This may lead to irreversible damage and even death. Superoxide dismutase (SOD) and catalase (CAT) are antioxidant enzymes responsible to reduce ROS levels [40]. A more active metabolism will also have energetic needs, and isocitrate and lactate dehydrogenases (IDH and LDH, respectively) are important biomarkers to pinpoint if the organism is favoring aerobic or anaerobic metabolism for energy acquisition [41]. Moreover, at the energetic level, the measurement of the electron transport system (ETS) activity will inform the increase of cellular metabolism and energetic expenditure (energy consumption; Ec), while, by measuring lipid, carbohydrate, and protein contents the energy available is known (Ea) [42]. The ratio between the Ea and the Ec will give the cellular energy allocation (CEA) at a certain period of time, indicative if the stress is conditioning the organisms’ energy [42]. These biomarkers, in addition to the mechanistic information given, will also pinpoint potential trade-offs and ecological consequences [43].

The present study aims to predict the effects of a summer heat wave on competing native and non-native invasive congener bivalve species, by comparing their intrinsic fitness and overall activity, based on multilevel responses (bioturbation, nutrient generation, and subcellular biomarkers, including oxidative stress and energetic biomarkers). This will contribute to understanding and comparing the impacts of a heat wave scenario on a native vs invasive bivalve species, addressing: (a) if a summer heat wave will have more severe impacts in the native species ecological engineering behavioral endpoint compared to the invasive species, and/or (b) the heat wave will induce more oxidative stress and energetic expenditure in the native species that may result in trade-offs that will potentially make the invasive outperform the native species. 

## 2. Materials and Methods

### 2.1. Species and Aquaria Setup

Both native *Ruditapes decussatus* and invasive *R. philippinarum* were supplied by fishermen who harvested them in shellfish banks in Ria de Aveiro (40°37′00″ N; 8°44′27″ W), a shallow, mesotidal, coastal lagoon in the north-western coast of Portugal—*R. decussatus* size was 41.52 ± 0.63 mm, and *R. philippinarum* 32.41 ± 0.65 mm (individual mean width ± SE), corresponding to 15.70 ± 0.45 g and 8.86 ± 0.16 g (wet weight ± SE), respectively. Biomass in each experimental aquarium (12 × 12 × 35 cm, internal dimensions) was adjusted to be as close as possible by manipulating densities; biomass was calculated after Brey [44], which are close to those found in natural environment [32]. Before the experimental procedures, specimens were acclimated in the laboratory for 7 days, at 17.5 °C and salinity 28, in 6 acclimation tanks (35 × 50 × 28 cm, with ~8 cm of sand, overlain with ~26 L of seawater diluted with ultrapure water). The acclimation tanks were aerated, and 1/3 of the seawater was replaced every other day to avoid the accumulation of toxic nitrogen compounds in the water. During the acclimation period, specimens were fed with a solution of dried mixed green microalgae (Phytobloom Reef Feed, Necton: *Nannochloropsis* sp., 45%, *Tetraselmis* sp., 45% and *Isochrysis* sp. 10%, ~1 µg per g of bivalve wet weight) every other day, after the water replacement. Each experimental aquarium was filled up to 10 cm with sand (medium sand (82.7%) [45]; organic matter loss on ignition (450 °C, 8 h) = 0.18 ± 0.008% mean ± SE), previously air dried for 3 weeks to avoid the introduction of unwanted living macrofauna, and overlain to a total of 30 cm with sand-filtered, UV-treated, seawater (~3 L; adjusted with ultrapure water at salinity 28). Before the beginning of the experimental period, the water was replaced to avoid nutrient pulses associated with assembly [22]. 

The experiments run for 12 days, within a temperature-controlled room under a 9:15 light:dark regime. Both species were exposed to 2 different temperature treatments: (a) constant temperature (CT; 17.5 °C, which is close to the average water temperature in Ria de Aveiro (17–18 °C) [46,47,48]) and (b) heat wave simulation (HW; 6 days at 17.5 °C, followed by 6 days at 22 °C). Present simulated MHW fits the definition provided by Hobday et al. [16] as a period of at least 5 consecutive days when the temperature is above the 90th percentile. This includes several climatic events that occurred in the Iberian Peninsula in recent decades [13]. The increase in temperature in HW aquaria was achieved with submersible heaters (Jäger 3612, EHEIM, Deizisau, Germany). The HW temperature was achieved within the day. To avoid block effects, the experimental aquaria were randomised using the *block.random* function of the package *psych* [49] in R statistical and programming environment [50]. Pumped air for each aquarium ensured aeration and water circulation. Physicochemical parameters (salinity, temperature, pH, and dissolved oxygen), as well as mortality, were recorded every other day. Therefore, the experimental design consisted of 28 aquaria for the following approach: at day 6 (D6), before the temperature elevation, bivalves from 4 aquaria per species (*R. decussatus*: n = 12; *R. philippinarum*: n = 16; 8 aquaria in total) were recovered and immediately frozen at −80 °C for subsequent determination of biochemical parameters (8 aquaria in total); at day 12 (D12), at the end of the HW, and integrating the subcellular effects of the previous 6d, individuals from 4 aquaria per species and per temperature treatment (*R. decussatus*: n = 24; *R. philippinarum*: n = 32; 16 aquaria in total) were recovered as explained above. Four control aquaria, without bivalves, were also included and kept at CT until the end of the experiment, for procedural control. Individuals were measured and weighed at the beginning and at the end of the experiment. Bivalves were not fed during the experimental period. 

The concentrations of nutrients (NH_3_-N, and PO_4_-P) in the water column were analysed from aliquots (25 mL·aquaria^−1^, 0.45 µm filtered) taken at D6 and D12, using standard photometric methods [51]. 

### 2.2. Measurement of Particle Reworking 

The f-SPI (fluorescent sediment profile imaging [22,26] method was used to assess, non-invasively, the amount of particle reworking. Luminophores (30 g; dyed sediment particles that fluoresce under UV light, 125–250 μm diameter, orange colour; Brian Clegg Ltd., Rochdale, UK) were added to each experimental aquarium, before the introduction of the bivalves. At D12, each aquarium was photographed on all sides under UV light with a Canon EOS 7D reflex digital CMOS camera (18.0 megapixels, set for 0.5 s exposure, f/2.8 diaphragm aperture, ISO 800 film speed equivalent), revealing the luminophores’ distribution. Images (JPEG, RGB colour) were cropped to the full internal width of the aquaria (12 cm) and all 4 sides’ photographs were merged (48 cm = 5396 pixels, effective resolution = 88.9 μm per pixel). Images were analysed with a custom-made plugin that runs within ImageJ (Version 1.51j8, US National Institute of Health, available at http://imagej.nih.gov/ij/, accessed on 10 February 2017). This plugin converts images to a binary data matrix of the distribution and occurrence of luminophore pixels (0 = background sediment, 1 = luminophore), and returns: (a) mean (^f-SPI^L_mean_, time-dependent indication of mixing), (b) median (^f-SPI^L_median_, typical short-term depth of mixing), and (c) maximum (^f-SPI^L_max_, maximum extent of mixing integrated over the long term) mixed depths of particle redistribution, as output. The distance between the highest and lowest points of elevation along the sediment-water interface, (d) surface boundary roughness (SBR = highest − lowest), was also assessed, providing a proxy for surficial faunal activity. Surface boundary roughness, for each aquarium, was averaged from all four sides. These are descriptors of faunal mediated sediment particle reworking, that translates, to some extent, different aspects of invertebrate behaviour [52].

### 2.3. Determination of Biochemical Responses and Data Treatment

Subcellular biomarker analyses were performed in whole-body soft tissues of both native (*R. decussatus*) and non-native (*R. philipinarum*) clams. Each individual was homogenised (Ystral, Ballrechten-Dottingen, Germany) in cold potassium-phosphate buffer (0.1 M; pH 7.4) following a 1:10 proportion (m:v). All aliquots were preserved at −80 °C until further analysis.

Post-mitochondrial supernatant (PMS) was isolated through differential centrifugation (10,000× *g*, 20 min, 4 °C), and used in further measurements of superoxide dismutase (SOD) activity [53], catalase (CAT) activity [54], and protein content (for normalization purposes) following Bradford’s method adapted to microplate [55]. In an additional supernatant (3000× *g*, 5 min, 4 °C), activities of isocitrate dehydrogenase (IDH) (method by Ellis and Goldberg [56], adapted by Lima et al. [57]) and lactate dehydrogenase (LDH) [58,59], were measured. Electron transport system activity (ETS) was analysed according to the method of King & Packard [60], modified by De Coen & Janssen [42], on mitochondrial supernatant (1000× *g*, 10 min, 4 °C). The remaining homogenate was separated to later quantify: DNA damage (DNAd) [61], lipid peroxidation (LPO) [62], and Energy Reserves—total protein (P), carbohydrates (C), and lipid (L) contents [42,63]. Cellular energy allocation (CEA) [64], a proxy on energy trade-offs, was calculated as: CEA = *Ea*/*Ec*, where *Ea* (available energy) = carbohydrate  +  lipid  +  protein, and *Ec* (energy consumption) = ETS activity. 

All biomarker spectrophotometric measurements of each sample were analysed in technical triplicates at 25 °C, using a Synergy H1 Hybrid Multi-Mode Microplate Reader (BioTek Instruments, Winuski, VT, USA). Potassium-phosphate homogenization buffer (0.1 M; pH 7.4) was used as blank in all assays. 

To allow the direct comparison of responses between species, the biomarker results for each temperature condition at the end of the exposure (*D*12), for both species, were normalised as % of change in relation to the *D*6 biomarker values of the respective species [65], as follows:

(1) Bi,jn=Bi,jD12−B¯iD6B¯iD6∗100

where $B_{i,j}^{n}$ is the normalized biomarker value for species *i* and temperature treatment *j*, $B_{i,j}^{D12}$ is the biomarker value on *D*12 for species *i* and temperature treatment *j*, and $\;{\bar{B}}_{i}^{D6}$ is the average value for the biomarker on *D*6 for species *i*. 

Biomarkers results were further integrated under the Integrated Biological Response version 2 (IBR_v2) index, after Sanchez et al. [66]. This index represents the stress levels and overall impact of each experimental condition*species, and in this study, the integrated biomarker responses at *D*12 (CAT and SOD activities, LPO, DNAd, LDH, IDH, and CEA) were compared to its reference value (*D*6), retrieving an induction or inhibition value, based on the reference deviation concept. For that, each individual biomarker value was firstly log transformed to reduce variation

(2) Yi,j=logBi,jD12B¯iD6

where $Y_{i,j}$ = individual log-value for species *i* and temperature treatment *j*. Then, the general mean of each biomarker (µ) and standard deviation ($s_{i,j}$) of $Y_{i,j}$ was calculated, for species *i* and temperature treatment *j*, and each $Y_{i,j}$ standardized as

(3) $$Z_{i,j}=\left(Y_{i,j}-\mu \right)/s_{i,j}$$

For the calculation of a biomarker deviation index, the biomarker data of D6 was also standardized as $Z_{i}^{D6}={\bar{Y}}_{i}^{D6}$ where, for each species *i*,

(4) YiD6=logBiD6B¯iD6

The biomarker deviation index ($A_{i,j}$) was then calculated for each biomarker, for species *i* and temperature treatment *j*, as $A_{i,j}=Z_{i,j}^{D12}-Z_{i}^{D6}$. Finally, IBR_v2 was assessed as the sum of all absolute values of biomarker deviation indexes, for each biomarker *b*:

(5) $$IBRv2=\sum^{\;}|A_{i,j,b}|$$

### 2.4. Statistical Analysis

Independent regression models for the dependent ecological variables (particle reworking: SBR, ^f-SPI^L_mean_, ^f-SPI^L_median_, and ^f-SPI^L_max_; nutrient concentration: NH_3_-N and PO_4_-P) and the normalized biomarkers values (oxidative stress: SOD activity, CAT activity, DNAd, and LPO; energetic metabolism: IDH and LDH activities; energy allocation: ETS activity, lipid content, carbohydrate content, protein content, total energy available, and cellular energy allocation) against the full factorial combination of independent variables (temperature treatment and species as fixed terms) were tested for statistical significance of their terms. As our focus was to establish the effects of different species, rather than presence versus absence effects, data from the procedural control (aquaria without animals) was removed from the statistical analysis of the ecological effects (bioturbation and nutrients) [22,67,68]. The models were extended to include the appropriate variance covariate structure using a generalized least squares (GLS) estimation procedure [69] (minimal adequate model summaries are appended as Supplementary Material). This extension allows unequal variance along explanatory variables and avoids data transformation when heteroscedasticity is verified. The adequate variance covariate structure was established by comparison with the initial regression model without a variance covariate structure, using a restricted maximum-likelihood (REML) estimation, and based on Akaike Information Criteria (AIC) and visual comparisons of residuals plots. The fixed structure of the minimal adequate model was determined by backward selection based on the maximum-likelihood (ML) ratio test, and re-expressed using REML to obtain the numerical output [70]. The data analyses were carried out within the R statistical and programming environment [50] and the package *nlme* [71]. Plots were made with *ggplot2* package [72]. A significance level of 0.05 was considered in all test procedures. 

## 3. Results

### 3.1. Environmental Variables

The experimental physicochemical conditions are included in Table 1. The recorded values showed minor variations between treatments. Nevertheless, pH showed an overall increase after D6; dissolved oxygen showed a decrease in those aquaria that were actively heated. Water temperature also increased after D6 in CT aquaria, by thermal influence from the HW aquaria. Despite being randomly distributed, the aquaria were close enough for heat to affect the CT, which were dependent on the room temperature. However, the difference between HW and CT, after D6, was still higher than 4 °C (HW: 22.13 ± 0.15 °C; CT: 18.12 ± 0.05 °C (mean ± SE)).

### 3.2. Behavioural and Ecological Responses 

#### 3.2.1. Sediment Reworking

**Figure 1:**
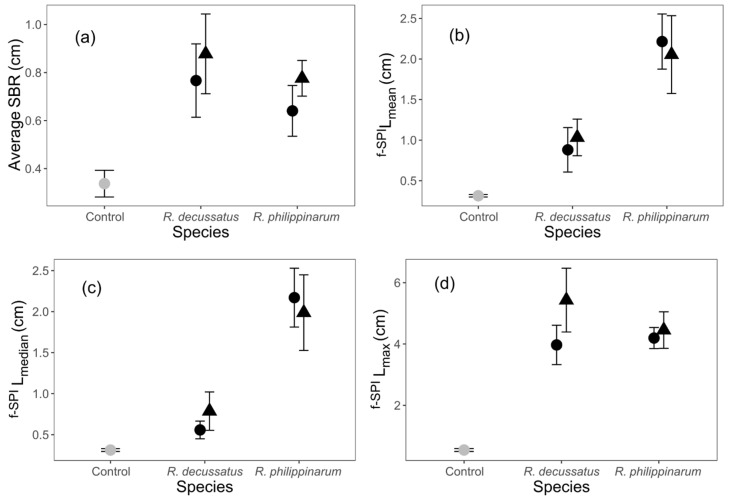
The effects of different temperature treatments on the sediment reworking by *Ruditapes decussatus* and *R. philippinarum* (cm, mean ± SE): (**a**) Surface boundary roughness (SBR), (**b**) mean mixed depth of luminophores’ redistribution (^f-SPI^L_mean_), (**c**) median mixed depth of luminophores’ redistribution (^f-SPI^L_median_), and (**d**) maximum mixed depth of luminophores’ redistribution (^f-SPI^L_max_), at day 12. For clarity, jitter has been applied to the × = argument of the plot function to avoid overplotting. For comparison, measurements in the absence of animals are presented (grey, control). 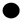 constant temperature; 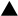 heat wave; n = 20.

The statistical significance and minimal adequate models’ summaries are appended as Supplementary Material (“Supplemental material: Statistical significance and minimal adequate model summaries”). The heat wave temperature treatment did not affect any sediment reworking measurement. There were no significant differences between levels in each factor, for surface boundary roughness (SBR) and maximum luminophores depth (^f-SPI^L_max_) (Supplemental Table S1, Figure 1d). There were differences between species for both mean (^f-SPI^L_mean_: df = 2, L-ratio = 11.074, *p* = 0.004; Supplemental Material Model S1) and median (^f-SPI^L_median_: df = 2, L-ratio = 12.528, *p* = 0.002; Model S2) luminophore depths, and, for both measurements, values were higher for *R. philippinarum* (Figure 1b,c; *R. philippinarum* ^f-SPI^L_mean_ = 2.13 ± 0.27 and ^f-SPI^L_median_ = 2.08 ± 0.77, vs *R. decussatus* ^f-SPI^L_mean_ = 0.96 ± 0.17 and ^f-SPI^L_median_ = 0.67 ± 0.13 (cm ± SE)). 

#### 3.2.2. Nutrients

**Figure 2:**
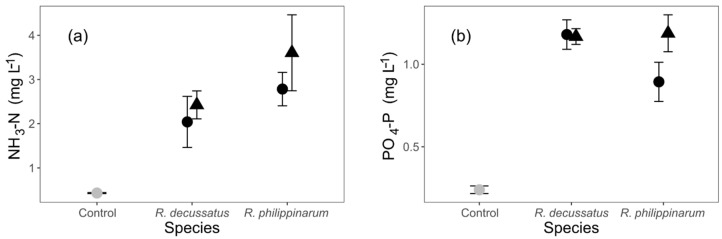
The effects of different temperature treatments and species (*Ruditapes decussatus* and *R. philippinarum*) on (**a**) NH_3_-N and (**b**) PO_4_-P concentrations (mg L^−1^) in the water at day 12. For clarity, jitter has been applied to the × = argument of the plot function to avoid overplotting. 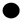 constant temperature; 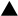 heat wave; n = 20.

For both analysed nutrients, NH_3_-N and PO_4_-P, there were no significant differences between any tested levels at D12 (Supplemental Material: Supplemental Table S1, Figure 2).

### 3.3. Subcellular and Biochemical Responses 

Statistical differences were found in the relative change of almost all tested subcellular and biochemical responses: these were often due to differences between the two species (statistical significance and minimal adequate models’ summaries in Supplementary Material). Yet, temperature treatment affected the variation on some of the energy metabolism-related measurements, in interaction with species identity. Nevertheless, IBR responded only to temperature treatment.

#### 3.3.1. Oxidative Stress and Damage 

**Figure 3:**
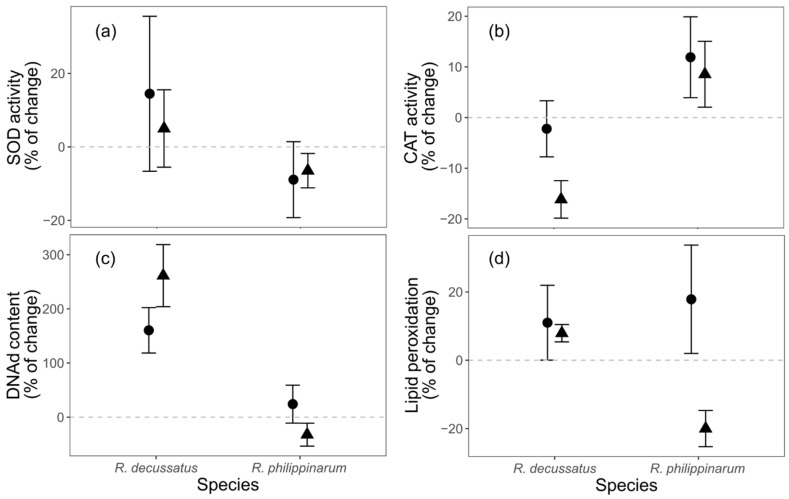
The effects of different temperature treatments on the relative change in oxidative stress biomarkers of *Ruditapes decussatus* (n = 24) and *R. philippinarum* (n = 32): (**a**) superoxide dismutase (SOD) activity, (**b**) catalase (CAT) activity, (**c**) DNA damage (DNAd), and (**d**) lipid peroxidation (LPO). For clarity, jitter has been applied to the × = argument of the plot function to avoid overplotting. 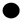 constant temperature; 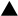 heat wave.

The variation in oxidative stress and related damage biomarkers, from D6 to D12, was significantly affected by species identity, except for superoxide dismutase (SOD: Figure 3a). Catalase (CAT) activity variation was affected only by species (Supplemental Table S2 and Model S3; Figure 3b) and generally with a decrease in activity for *R. decussatus* (−8.56 ± 3.70% ± SE) and increase for *R. philippinarum* (10.5 ± 5.29% ± SE). While non-significant, in *R. decussatus* this decrease in CAT activity was even more evident when under the heat wave (HW) temperature treatment (CT: −2.220 ± 5.534; HW: −16.161 ± 3.707. (% ± SE)). The variation in DNA damage (DNAd) was affected by species identity (Supplemental Table S2 and Model S4; Figure 3c), with very high values in *R. decussatus* (206.326 ± 35.575% ± SE) and close to zero in *R. philippinarum* (0.724 ± 22.585% ± SE). Lipid peroxidation (LPO) was again affected by species (Supplemental Table S2 and Model S5; Figure 3d). Yet, despite the interaction between species and temperature treatment being non-significant (d.f. = 2, L-ratio = 3.045, *p* = 0.081), LPO variation on *R. philippinarum* was negative (i.e., there was a reduction in LPO from D6 to D12) when under HW (−19.991 ± 5.303;% ± SE), while the other factorial combinations where all positive and within a narrow range (*R. decussatus*|CT: 10.993 ± 10.963; *R. decussatus*|HW: 7.914 ± 2.557; *R. philippinarum*|CT: 17.845 ± 15.873 (% ± SE)). 

#### 3.3.2. Energy Metabolism Related Enzymes

**Figure 4:**
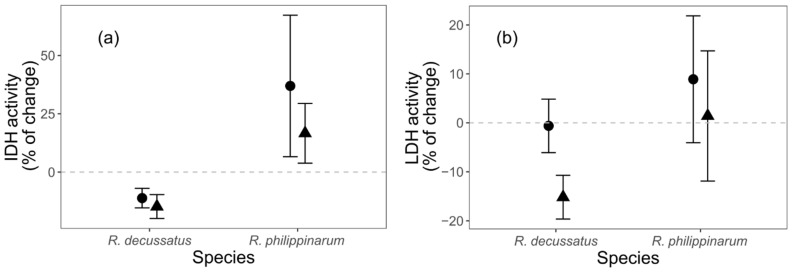
The effects of different temperature treatments on the relative change in energetic metabolism related enzymes of *Ruditapes decussatus* (n = 24) and *R. philippinarum* (n = 32): (**a**) isocitrate dehydrogenase (IDH) activity, and (**b**) lactate dehydrogenase (LDH) activity. For clarity, jitter has been applied to the × = argument of the plot function to avoid overplotting. 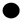 constant temperature; 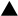 heat wave.

Regarding energy metabolism related biomarkers, only isocitrate dehydrogenase (IDH) presented significant differences (Supplemental Table S2), with species identity as the driving factor. From D6 to D12, *R. decussatus* had a decrease in IDH levels (−12.761 ± 3.193% ± SE), while it increased in *R. philippinarum* (28.479 ± 18.292% ± SE) (Figure 4a; Model S6). The invasive species held lower IDH activity levels when under the HW treatment (Figure 4a). 

#### 3.3.3. Cellular Energy Allocation

**Figure 5:**
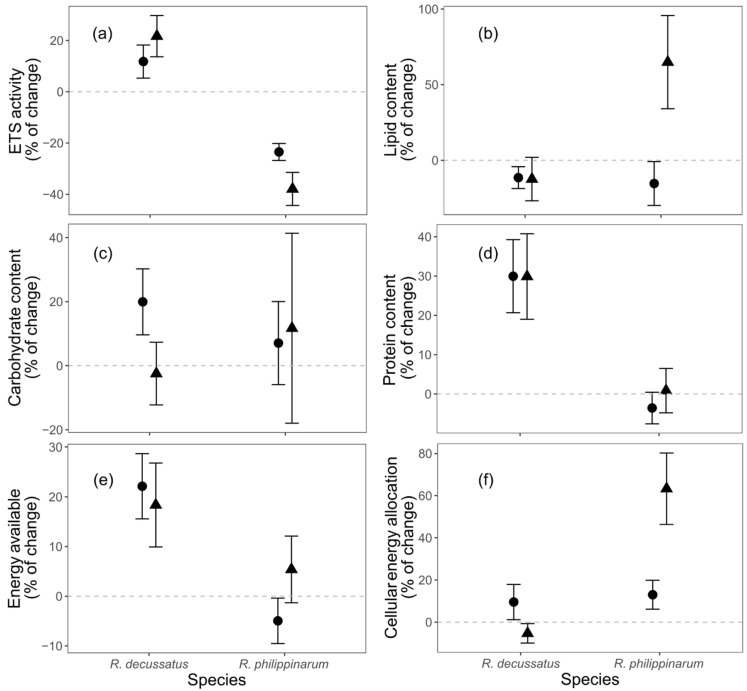
The effects of different temperature treatments on the relative change in energy allocation biomarkers of *Ruditapes decussatus* (n = 24) and *R. philippinarum* (n = 32): (**a**) electron transport system (ETS) activity, (**b**) lipid, (**c**) carbohydrate, and (**d**) protein contents, (**e**) energy available, and (**f**) cellular energy allocation. For clarity, jitter has been applied to the × = argument of the plot function to avoid overplotting. 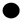 constant temperature; 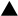 heat wave.

Electron transport system (ETS) activity was affected by the interactive effects of both factors (Supplemental Table S2 and Model S7). The factor species was the most influential variable (df = 2, L-ratio = 42.434, *p* <.0001), followed by temperature (df = 2, L-ratio = 4.503, *p* = 0.105). *Ruditapes decussatus* showed a positive variation (an increase from D6 to D12), which was enhanced by the HW treatment (CT: 11.793 ± 6.452; HW: 21.707 ± 8.037 (% ± SE)), while in *R. philippinarum* the shift was negative (decreasing from D6 to D12), with HW treatment responsible for the lowest values (CT: −23.482 ± 3.293; HW: −37.918 ± 6.457 (% ± SE)) (Figure 5a). Similarly, lipid content (Figure 5b) was affected by the interaction species x temperature treatment (Supplemental Table S2 and Model S8). The factor species was again the most influential one (species: df = 2, L-ratio = 8.345, *p* = 0.015; temperature treatment: df = 2, L-ratio = 6.361, *p* = 0.042). The variation in lipid content from D6 to D12 was always negative, without significant differences (*R. decussatus*|CT: −11.466 ± 7.196; *R. decussatus*|HW: −12.442 ± 14.347; *R. philippinarum*|CT: −15.354 ± 14.535 (% ± SE)), except for *R. philippinarum* under HW (*t*-value = 2.347, *p* = 0.024; 64.926 ± 30.826% ± SE). 

Carbohydrate content variation (Figure 5c) had no significant differences between treatments, with positive variation in almost every factorial combination (*R. decussatus*|CT: 19.954 ± 10.308; *R. philippinarum*|CT: 7.068 ± 12.984; *R. philippinarum*|HW: 11.705 ± 29.663 (% ± SE)), except for *R. decussatus*|HW, which showed a decrease (−2.462 ± 9.776% ± SE). Protein content (Figure 5d) was different between species (Supplemental Table S2 and Model S9). *Ruditapes decussatus* suffered a positive increase in protein content from D6 to D12 (29.925 ± 6.908% ± SE), while *R. philippinarum* showed a slight decrease (−1.718 ± 3.275% ± SE). Energy available, the sum of protein, carbohydrates, and lipids followed the same tendency as total protein content, with significant differences between species (Supplemental Table S2 and Model S10; Figure 5e). It increased on *R. decussatus* (20.424 ± 5.137% ± SE) and showed a small decrease in *R. philippinarum* (−0.642 ± 3.923% ± SE). The cellular energy allocation (Figure 5f) changes from D6 to D12 was again influenced by the interactive effects of both factors (Supplemental Table S2 and Model S11), with species as the most influential factor (species: df = 2, L-ratio = 10.326, *p* = 0.006; temperature treatment: df = 2, L-ratio = 8.882, *p* = 0.012). While there were no differences between both species at CT (*R. decussatus*: 9.554 ± 8.374; *R. philippinarum*: 13.003 ± 6.843 (% ± SE)), the HW had contrasting effects on both species (*t*-value = 3.912, *p* = 0.000), with a decrease in *R. decussatus* (−5.310 ± 4.598; % ± SE) and a prominent increase in *R. philippinarum* (63.281 ± 16.921; % ± SE). 

#### 3.3.4. Integrated Biological Response

**Figure 6:**
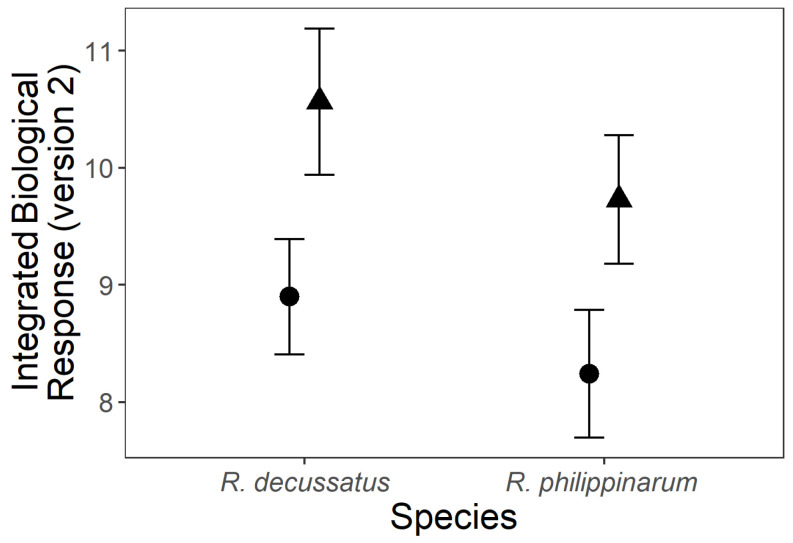
The effects of different temperature treatments on the Integrated Biological Response (version 2) of *Ruditapes decussatus* (n = 24) and *R. philippinarum* (n = 32). For clarity, jitter has been applied to the × = argument of the plot function to avoid overplotting. 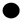 constant temperature; 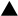 heat wave.

**Figure 7:**
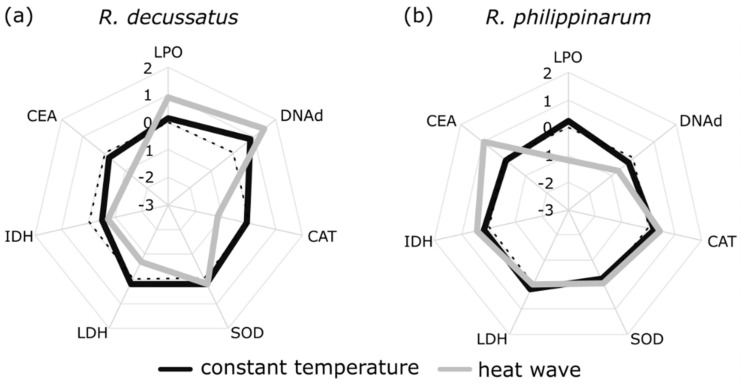
Star plots integrating all biomarkers measured in (**a**) *Ruditapes decussatus* (n = 24) and (**b**) *R. philippinarum* (n = 32) at constant temperature and after a heat wave simulation: SOD—superoxide dismutase activity; CAT—catalase activity; DNAd—DNA damage; LPO—lipid peroxidation levels; IDH—isocitrate dehydrogenase activity; LDH—lactate dehydrogenase activity; and CEA—cellular energy allocation (which incorporates data on lipid, protein, and carbohydrate contents and electron transport system levels).

The integrated biological response (version 2) (IBR_v2, Figure 6) was significantly affected by temperature (Supplemental Table S2 and Model S12), with higher values found under the HW treatment (CT: 8.543 ± 0.369; HW: 10.146 ± 0.417 (% ± SE)). However, the differences between species and temperature treatments are visible in the integrative star plots for all measured biomarkers (Figure 7), where the contribution of each biomarker for IBR is detailed. Again, the HW treatment affected both species, while at CT the normalized values are close to zero. Yet, the HW was responsible for contrasting changes in different biomarkers according to the species. Under the HW, in the native *R. decussatus* (Figure 7a) a reduction on CEA and on CAT and LDH activities, along with an increase in oxidative damage (LPO and DNAd), are the main responses influencing the IBR index. In opposition, for the invasive *R. philippinarum* under HW (Figure 7b), the driving responses are a reduction on LPO and DNA damage levels along with an increase on CEA. These observations are in line with the results of the statistical analysis of individual biomarkers.

## 4. Discussion

While both species differed in the intensity and depth of particle reworking, this ecological process did not suffer a differential impact as a consequence of the heat wave (HW). Differences in the luminophore depths between both species may be partially explained by body size differences. Nevertheless, the larger *R. decussatus* showed smaller mean and median luminophore depths. It should be noted that, however, *R. philippinarum* held higher densities, which could have contributed to a higher level of intraspecific competition and subsequent avoidance behaviour. With *R. philippinarum* featuring shorter siphons [73], the number of upward and downward movements within the sediment might also have been higher than those of *R. decussatus*. Adding to this, smaller individuals may show higher metabolic requirements [74], and therefore an increased active search for food. These concur to increase mean and median sediment reworking depths, which are proxies for the intensity of reworking [52]. A similar tendency was verified in another invasive bivalve, *Corbicula fluminea*, with smaller individuals responsible for higher bioturbation levels [22]. Surprisingly, maximum luminophore depths were similar on both *Ruditapes* species, which means that, despite size, density, or siphon length, in the long term both species might reach similar levels of particle reworking, and therefore, differ mostly in the rate of this process. The phylogenetic/taxonomic proximity of both species was expressed clearly in the behavioral response to thermal stress, which is absent in both cases, and therefore not conveyed into ecosystem functioning, under the specific set of conditions under analysis. The results of nutrient content in the water column reinforces this lack of expression at the ecological level, as responses showed no statistical significance, even if densities were slightly biased towards *R. philippinarum*.

Despite the absence of response on both species to the HW treatment at the ecological and behavioral levels, there were significant responses to thermal stress at the subcellular levels for both species. The analysis of subcellular biomarkers can act as an early warning tool for later-in-time responses in higher level of biological organization, which may occur after the stress exposure stage [21], and not depicted at this stage. Addressing the used ecological endpoints later-in-time, along with others such as growth and reproduction, would provide critical additional information about this potential link of biomarker responses with ecological processes and populational dynamics, and the expected trade-off that give advantage to a certain species [22], and that give relevance to the early-in-time responses. The use of normalized values, with biomarker levels from D6 as baseline (prior to the heat wave simulation), proved to be a useful approach in this context and allowed comparison of the relative changes in response to stress between species. Contrasting biomarker responses were observed, with lower impacts in the invasive species under the HW. The native *R. decussatus* showed a higher level of oxidative damage, as consequence of a compromised antioxidant and energetic investment given by the reduction on catalase and lactate dehydrogenase activities, while also showing a reduction in their cellular energy allocation. On the other hand, the invasive *R. philippinarum* seems to benefit from the increase in the temperature by showing a reduction in damage (lower lipid peroxidation and DNA damage), and a reinforcement of cellular energy allocation. The increase in the cellular energy allocation index, which relates energetic reserves to their consumption by cellular metabolism [42], suggests that the invasive species may have a higher energy budget for other functions, such as growth, reproduction, and locomotion/burrowing activity. Due to the defined nature and length of the experiment, growth and reproduction were not assessed but one cannot exclude that these endpoints could be reactive at a later period and translate the trade-offs better than the present studied higher level of organization proxies (behaviour). The increase in cellular energy allocation in *R. philippinarum* is justified mainly by the increase in lipid content, together with a lesser energetic expenditure, as electron transport system values reflect. One must add that, despite no fresh food being added to the water during the experiment, the sediment was not depleted of organic matter and detritus on which both species may have fed on to alter energy reserves [32]. One may also not disregard that the unexpected increase in lipids may also be related to an eventual *r*-strategy from the invasive species, with stress triggering gonad maturations and oocyte formation and the biosynthesis of lipids [75,76,77], these lipids being the main reserve of these structures [78,79]–while the native species relied on a *k-*strategy, as previously reported for other native and invasive bivalves under stress [5]. After integrating all these responses into the integrated biological response index, it becomes clearer that the HW treatment induced alterations in the biochemical stress responses for both species. However, the biomarker responses contributing to the higher index in both species are opposite, indicating a negative impact to the native species (elevated damage levels and lower energetic investments and cellular energy allocation) while those in the IS indicate that the individuals are coping better with the challenge (less damage and higher cellular energy allocation). In sum, these results suggest that, under the same HW conditions, the IS will possess a better condition, which might provide the marginal gains that makes non-native species better contenders in interspecific competition.

There is reduced experimental evidence in the literature that physiological tolerance to multiple environmental conditions is an obligatory characteristic of IS [4,19,80]. However, Zerebecki and Sorte [19] verified a positive link between the geographic range (and the maximum habitat temperature) and the temperature tolerance of epibenthic communities in California (USA), and simultaneously found that invasive species withstood higher lethal temperatures. In the same study, the authors found that the expression of a heat-shock protein was higher in the invasive tunicate *Diplosoma* than in the native *Distaplia*. For bivalves, a similar tendency was found in two *Mytilus* species: the invasive *M. galloprovincialis* showed higher expression of heat-shock proteins, with a higher induction temperature threshold, than the native *M. trossulus* [81]. Another freshwater bivalve study showed similar results, with the invasive *Sinanodonta woodiana* dealing better with higher temperatures and contamination levels than the native *Anodonta anatina* [4]. Sorte et al. [7] also analysed the differential survival between native and non-native species in a marine fouling community, and found that non-native had higher LT_50_ values (~2 °C average), which could indicate that these species can cope with higher temperature levels and show sublethal responses later during a thermal anomaly. Simultaneously, the same authors predicted abundance and growth rates increases for most tested IS, under a +4.5 °C temperature increase scenario. All of these are examples of how temperature mediates the survival and success of non-native IS. However, the idea that IS are favored by eury-tolerance, as the capacity to withstand wider ranges of environmental stressors [30], could be challenged. The concept of eury-tolerance includes, for example, tolerance to wide ranges of salinity, temperature, or contaminants [4,30,82], offering competitive advantages towards their native counterparts, particularly in transitional aquatic systems such as estuaries. Nevertheless, the hypothesis of eury-tolerance in IS mostly assumed due to wide geographical ranges as a surrogate measure for extended ranges of temperature or salinity (or other environmental variables). Despite conflicting reports on the tolerance range of IS, global climate change is often reported as an invasion facilitator [1,83], especially because species from warmer regions can expand their geographic ranges to higher latitudes [7], where they can now find more suitable conditions than before. For instance, the native *R. decussatus* showed a wider tolerance range for salinity than both the invasive *R. philippinarum* and the native *Venerupis corrugata* [23]. Other examples are described by McMahon [80] who reviewed the physiological adaptations in aquatic invasive invertebrates and found that the high-profile bivalve invaders *Corbicula fluminea* and *Dreissena polymorpha* showed reduced resistance and capacity adaptations (with massive mortality rates under extreme environmental conditions) when compared with endemic native unionoideans. This author sustains that the species invasive prowess is mostly related to *r-*selected traits (e.g., fast population growth, early maturity, and high fecundity) while native species must rely on their resistance capacity to warrant maintenance under natural disturbance, due to the *k-*selected traits. Ferreira-Rodríguez and colleagues [5] reached similar conclusions for the invasive *C. fluminea* vs the native *Unio delphinus*. A similar trend was found within a group of freshwater pecarid crustaceans [84]. In the present study, results show that *R. philippinarum* was less affected than the native *R. decussatus*. This means that, even without proving the extended tolerance range to temperature, i.e., eury-thermality [30], one can hypothesize that the top limit of the thermal range of the IS *R. philippinarum* is higher than that of its native counterpart *R. decussatus*.

One of the implications of the results here addressed is that the current climate crisis, not only due to the increase in average temperature, but especially due to the increase on the number of heat waves [6,17], may favor this particular IS, which is already a concern due to human-mediated dispersion as valued species [3,8,33]. It is unclear if the first decline in native species was due to the Manilla clam introduction in the Portuguese territory—which occurred almost simultaneously with the first signs of that decline—or if other factors, such as overfishing, diseases, or habitat modification, might have occurred. In this context, nevertheless, results fit within the narrative that IS are indeed aided by the interactive effects of global changes—in this case, climate change and the human-mediated introduction of species—contributing to the homogenization of the biota [7,9]. This means that the synergy between climate change and human mediated dispersion contributes to *R. philippinarum* overriding the filters (dispersion and habitat suitability) that often control the establishment of introduced species [30,85,86], with an increase in the pressure on local communities [2,7]. Extreme temperature fluctuation is often accompanied by changes in dissolved oxygen and salinity, which interactions might have disproportionate effects on the bivalve’s metabolism and fitness and limit the ability to describe the effects of global changes solely based on thermal characterization. Because changes in ecosystem function due to an introduced species depend not only on the rate and intensity of ecosystem processes, but also on the interactions of the introduced species with the invaded community and the environment [25], there is a high level of unpredictability on the ecological consequences of such introduction. However, Lopes et al. [8], when comparing the same species, verified that the replacement of the native by the IS was not expressed as differences on bioturbation. This pair of species is also known to hybridize [31], so there are hints that the introduction and dispersion of this particular IS may drive small changes in ecosystem functioning, due to their phylogenetic proximity, supported by the concept of functional redundancy [1,68]. However, these genetic outcomes should be monitored for even more severe and unpredictable impacts. If the IS indeed favored by climate change, compositional changes in the invaded communities may also occur due to a disproportionate increase and consequential dominance that is often reported for several IS [2,7,19].

## 5. Conclusions

The present work intentionally studies a conservative heatwave, with a relative short period and little temperature range. However, considering that not only the number of HW is increasing, but also the duration of these phenomena, this also triggers the question as to whether longer, repeated, or more intense HW will convey different and pronounced results at the ecological and behavioural level among these species, measurable during the experiment timescale, or even if effects extend after the end of the HW. However, despite the reduced range that was evaluated here, the results from this work contribute to better enlighten the processes that might offer IS higher fitness and competitive advantages towards their native counterparts, supported by biomarker analysis as a set of early responding endpoints that represent potential trade-offs for those advantages later, even before the transposition into ecosystem processes and functioning. Present results indicate that the intensity of responses is not consistent at the different levels under analysis, which means that assumptions based on a single endpoint, and limited timeframe, may not provide enough information on the consequences of environmental stressors on the overall fitness of organisms, as usually the link between different levels of biological organization will emerge over time. Here, lower levels of biological organization, such as biomarkers, provide putative early-warning signs that should add a cautionary tale to the scenario, even when other individual level endpoints fail to depict differential effects. This underlines the relevance of integrative, multilevel studies to disclose the impacts of stressors, and particularly, in the scope of this study, thermal stress, in a context of competing species that share similar ecological niches.

## Figures and Tables

**Table 1:** Physicochemical conditions of the experimental units for each species (native *Ruditapes* *decussatus* and invasive *R. philippinarum)* and temperature treatments, before and after the introduction of the aquarium heaters at day 6 (mean ± SE). CT: constant temperature; HW: heat wave simulation.

		Salinity	Temperature (°C)	pH	Dissolved O_2_ (mg L^−1^)
Before D6	Control	28.14 ± 0.02	17.31 ± 0.07	8.08 ± 0.04	9.43 ± 0.08
	*R. decussatus*: CT	28.19 ± 0.02	17.41 ± 0.04	8.06 ± 0.03	9.27 ± 0.06
	*R. decussatus*: HW	28.18 ± 0.03	17.42 ± 0.06	8.07 ± 0.06	9.42 ± 0.05
	*R. philippinarum*: CT	28.17 ± 0.01	17.23 ± 0.04	8.08 ± 0.02	9.37 ± 0.05
	*R. philippinarum*: HW	28.19 ± 0.03	17.29 ± 0.06	8.10 ± 0.03	9.46 ± 0.05
After D6	Control	28.41 ± 0.06	18.18 ± 0.10	8.32 ± 0.01	9.41 ± 0.03
	*R. decussatus*: CT	28.53 ± 0.03	18.15 ± 0.09	8.29 ± 0.02	9.39 ± 0.07
	*R. decussatus*: HW	28.70 ± 0.09	21.90 ± 0.19	8.27 ± 0.04	8.64 ± 0.14
	*R. philippinarum*: CT	28.35 ± 0.04	18.03 ± 0.08	8.24 ± 0.01	9.27 ± 0.09
	*R. philippinarum*: HW	28.71 ± 0.13	22.35 ± 0.20	8.24 ± 0.02	8.27 ± 0.13

## Data Availability

Not applicable.

## Supplementary Materials

The following are available online at https://www.mdpi.com/article/10.3390/biology10121284/s1, Table S1: Summary of significant terms found in the generalised least squares models, Table S2: Summary of significant terms found in the generalised least squares models, Model S1: Mean Luminophore Depth (^f-SPI^L_mean_), Model S2: Median Luminophore Depth (^f-SPI^L_median_), Model S3: Catalase activity (CAT), Model S4: DNA damage (DNAd), Model S5: Lipid peroxidation (LPO), Model S6: Isocitrate dehydrogenase activity (IDH), Model S7: Electron transport system (ETS), Model S8: Lipid content, Model S9: Protein content, Model S10: Energy available (EA), Model S11: Cellular energy allocation (CEA), Model S12: Integrated biological response, version 2 (IBR_V2).
