# Supplementary material for: Does an Invasive Bivalve Outperform Its Native Congener in a Heat Wave Scenario? A Laboratory Study Case with Ruditapes decussatus and R. philippinarum
Source: Biology (Basel). 2021 Dec 7;10(12):1284. doi: 10.3390/biology10121284 (PMC8698865; doi:10.3390/biology10121284)
Supplement: Supplementary file 1 [file biology-10-01284-s001.zip › biology-1474581-supplementary.pdf]

Does an Invasive Bivalve Outperform Its Native Congener in a Heat Wave Scenario? A Laboratory Study Case with *Ruditapes decussatus* and *R. philippinarum*

Supplemental material: Statistical significance and minimal adequate model summaries

Daniel Crespo[1,2,3,*], Sara Leston[2], Lénia D. Rato[1], Filipe Martinho[2], Sara C. Novais[1], Miguel A. Pardal[2] and Marco F.L. Lemos [1,*]

[1] MARE—Marine and Environmental Sciences Centre, ESTM, Polytechnic of Leiria, 2520-641 Peniche, Portugal; lenia.rato@ipleiria.pt (L.R.); sara.novais@ipleiria.pt (S.C.N.)
[2] CFE - Centre for Functional Ecology—Science for People & the Planet, Department of Life Sciences, University of Coimbra, Calçada Martim de Freitas, 3000-456 Coimbra, Portugal; saraleston@ci.uc.pt (S.L.); fmdm@ci.uc.pt (F.M.); mpardal@uc.pt (M.A.P.)
[3] CIIMAR—Interdisciplinary Centre of Marine and Environmental Research, University of Porto, Terminal de Cruzeiros do Porto de Leixões, Avenida General Norton de Matos, S/N, 4450-208 Matosinhos, Portugal
* Correspondence: daniel.crespo@ipleiria.pt (D.C.); marco.lemos@ipleiria.pt (M.F.L.L.)

1) Statistical significance tables

Table S1. Summary of significant terms found in the generalised least squares models, using bioturbation components and nutrient concentrations as dependent variables and temperature treatment and species as explanatory variables.

Dependent variable	Significant terms	df	L-ratio	p
Particle reworking				
SBR	-	-	-	-
$^{f\text{-}SPI}L_{mean}$	Species	2	11.074	.004
$^{f\text{-}SPI}L_{median}$	Species	2	12.528	.002
$^{f\text{-}SPI}L_{max}$	-	-	-	-
Nutrients				
NH_3-N	-	-	-	-
PO_4-P	-	-	-	-

Table S2. Summary of significant terms found in the generalised least squares models, using the normalised subcellular and biochemical responses and the Integrated Biological Response (version 2) as dependent variables, and species (*Ruditapes decussatus* and *R. philippinarum)* and temperature (constant temperature and heat wave) as explanatory variables.

Dependent variable	Significant terms	df	L-ratio	p
Oxidative stress				
SOD activity	-	-	-	-
CAT activity	Species	2	8.852	.012
DNAd	Species	2	19.294	1e-04
LPO	Species	2	13.734	.001
Energy metabolism				
IDH activity	Species	2	7.158	.028
LDH activity	-	-	-	-
Energy allocation				
ETS activity	Species x Temperature	1	4.305	.038
Lipid content	Species x Temperature	1	5.539	.019
Carbohydrate content	-	-	-	-
Protein content	Species	2	13.943	9e-04
Energy available	Species	2	11.336	.004
Cellular energy allocation	Species x Temperature	1	8.508	.004
Integrative index				
IBR_V2	Temperature	2	7.961	.019

2) Minimal adequate models and coefficient estimations

Bioturbation

- Model S1: Mean Luminophore Depth (${}^{f\text{-}SPI}L_{mean}$)

$$ {}^{f\text{-}SPI}L_{mean} = f\,(species) $$

The minimal adequate model was a linear regression model with a GLS extension (*varIdent* variance structure), with *species* as variance covariate.

Minimal adequate model coefficient estimations for ${}^{f\text{-}SPI}L_{mean}$

	Value	S.E.	t-value	p-value
Intercept (*R. decussatus*)	0.957	0.167	5.742	0.000
R. philippinarum	1.178	0.321	0.321	0.003

- Model S2: Median Luminophore Depth (${}^{f\text{-}SPI}L_{median}$)

$$ {}^{f\text{-}SPI}L_{median} = f\,(species) $$

The minimal adequate model was a linear regression model with a GLS extension (*varIdent* variance structure), with s*pecies* as variance covariate.

Minimal adequate model coefficient estimations for ${}^{f\text{-}SPI}L_{median}$

	Value	S.E.	t-value	p-value
Intercept (*R. decussatus*)	0.673	0.127	5.302	0.000
R. philippinarum	1.407	0.301	4.677	0.000

Subcellular and biochemical responses

- Model S3: Catalase activity (CAT)

$$CAT = f\,(species)$$

The minimal adequate model was a linear regression model with a GLS extension (*varIdent* variance structure), with s*pecies* as variance covariate.

Minimal adequate model coefficient estimations for CAT

	Value	S.E.	t-value	p-value
Intercept (*R. decussatus*)	-8.557	3.704	2.310	0.026
R. philippinarum	19.055	6.459	2.950	0.005

- Model S4: DNA damage (DNAd)

$$DNAd = f\,(species)$$

The minimal adequate model was a linear regression model with a GLS extension (*varIdent* variance structure), with s*pecies x temperature* as variance covariate.

Minimal adequate model coefficient estimations for DNAd

	Value	S.E.	t-value	p-value
Intercept (*R. decussatus*)	194.617	34.596	5.625	0.000
R. philippinarum	-211.927	39.176	-5.410	0.000

- Model S5: Lipid peroxidation (LPO)

$$LPO = f\ (species)$$

The minimal adequate model was a linear regression model with a GLS extension (*varIdent* variance structure), with s*pecies x temperature* as variance covariate.

Minimal adequate model coefficient estimations for LPO

	Value	S.E.	t-value	p-value
Intercept (*R. decussatus*)	8.084	2.478	3.262	0.002
R. philippinarum	-24.954	5.718	-4.364	0.000

- Model S6: Isocitrate dehydrogenase activity (IDH)

$$IDH = f\ (species)$$

The minimal adequate model was a linear regression model with a GLS extension (*varIdent* variance structure), with s*pecies x temperature* as variance covariate.

Minimal adequate model coefficient estimations for IDH

	Value	S.E.	t-value	p-value
Intercept (*R. decussatus*)	-12.580	3.177	-3.960	0.000
R. philippinarum	32.356	12.138	2.666	0.011

- Model S7: Electron transport system (ETS)

ETS = f (species x temperature)

The minimal adequate model was a linear regression.

Minimal adequate model coefficient estimations for ETS; CT= constant temperature, HW= heat wave simulation

	Value	S.E.	t-value	p-value
Intercept (*R. decussatus;CT*)	11.793	5.814	2.029	0.049
R. philippinarum	-35.275	7.923	-4.452	0.000
HW	9.914	8.623	1.150	0.257
R. philippinarum x HW	-24.350	11.995	-2.030	0.049

- Model S8: Lipid content

Lipid content = f (species x temperature)

The minimal adequate model was a linear regression model with a GLS extension (*varIdent* variance structure), with s*pecies* as variance covariate.

Minimal adequate model coefficient estimations for lipid content; CT= constant temperature, HW= heat wave simulation

	Value	S.E.	t-value	p-value
Intercept (*R. decussatus;CT*)	-11.466	10.280	-1.115	0.271
R. philippinarum	-3.888	22.543	-0.172	0.864
HW	-0.976	15.247	-0.064	0.949
R. philippinarum x HW	81.256	34.619	2.347	0.024

- Model S9: Protein content

$$Protein\ content = f\ (species)$$

The minimal adequate model was a linear regression model with a GLS extension (*varIdent* variance structure), with s*pecies* as variance covariate.

Minimal adequate model coefficient estimations for protein content

	Value	S.E.	t-value	p-value
Intercept (*R. decussatus*)	29.925	6.908	4.332	0.000
R. philippinarum	-31.643	7.645	-4.139	0.000

- Model S10: Energy available (EA)

$$EA = f\ (species)$$

The minimal adequate model was a linear regression model.

Minimal adequate model coefficient estimations for EA

	Value	S.E.	t-value	p-value
Intercept (*R. decussatus*)	20.424	4.623	4.418	0.000
R. philippinarum	-21.065	6.400	-3.291	0.002

- Model S11: Cellular energy allocation (CEA)

$$CEA = f\,(species\ x\ temperature)$$

The minimal adequate model was a linear regression model with a GLS extension (*varIdent* variance structure), with s*pecies x temperature* as variance covariate.

Minimal adequate model coefficient estimations for CEA; CT= constant temperature, HW= heat wave simulation

	Value	S.E.	t-value	p-value
Intercept (*R. decussatus;CT*)	9.554	8.374	1.141	0.260
R. philippinarum	3.449	10.814	0.319	0.751
HW	-14.864	9.553	-1.556	0.127
R. philippinarum x HW	65.143	20.601	3.162	0.003

- Model S12: Integrated biological response, version 2 (IBR_V2)

$$IBR\_V2 = f\,(temperature)$$

The minimal adequate model was a linear regression model.

Minimal adequate model coefficient estimations for IBR_V2; CT= constant temperature, HW= heat wave simulation

	Value	S.E.	t-value	p-value
Intercept (CT)	8.544	0.368	23.229	0.000
HW	1.602	0.558	2.872	0.006
